# Bacterial type VI secretion system helps prevent cheating in microbial communities

**DOI:** 10.1093/ismejo/wrae003

**Published:** 2024-01-11

**Authors:** Alain Filloux

**Affiliations:** Singapore Centre for Environmental Life Sciences Engineering, Nanyang Technological University, 60 Nanyang Drive, 637551, Singapore; School of Biological Sciences, Nanyang Technological University, 60 Nanyang Drive, 637551, Singapore; Department of Life Sciences, Imperial College London, South Kensington Campus, London SW72AZ, United Kingdom

**Keywords:** T6SS, antibacterial toxin, microbial community, immunity, iron uptake

In their paper, “Trojan horse-like T6SS effector TepC mediates both interference competition and exploitative competition”, Song and colleagues (2024) remind us of an important societal principle that everyone contributes to benefit the collective population [1]. This challenging task is reflected in penal codes and civil rights, so that proper conduct and goodwill of all individuals serves the community as a whole. Although microbial “societies” do not have such rules codified, they nonetheless implement molecular strategies to restrict foes and cheaters within their respective populations and communities.

Microorganisms compete for resources, which can be scarce or poorly metabolizable. An ideal community will involve microorganisms with different capabilities, working synergistically to exploit available resources optimally [2]. Without synergy, and if coexistence requires competition, then bacteria have evolved strategies to fight [3], and ultimately the fittest will prevail. Bacterial competition can involve long-range or contact-dependent antagonism. Among long-range strategies, bacteria may release toxic substances that diffuse and kill other species. For example, *Pseudomonas aeruginosa* secretes a soluble pigment called pyocyanin [4], which has strong oxidative properties that eliminate *Staphylococcus aureus* and other bacteria. *Pseudomonas aeruginosa* can also release pyocins, that have various functions, including pore formation [5], that specifically target related species.

Our understanding of contact-dependent antagonism was enhanced by the discovery of the Type VI secretion system (T6SS) [6]. The T6SS is a complex molecular nanomachine, associated with the Gram-negative bacterial cell envelope, which can “fire” upon sensing danger, like encountering another bacterium. The T6SS can be compared to a crossbow firing poisoned arrows into microbial opponents, with the poison representing a cocktail of antibacterial toxins. These toxins are extremely diverse and can be peptidoglycan hydrolases, pore-forming proteins, phospholipases, nucleases, or NAD glycohydrolase and (p)ppApp synthase [7], compromising the pool of essential metabolites required for physiological integrity and cell function.

One caveat of toxin production is that the bacterial toxins being produced by the attacker must not “intoxicate” the source cell and also not harm neighbouring kin cells. For that reason, all antibacterial toxin genes are dependent on their associated immunity that diffuse the toxins through protein–protein interactions or by interfering with the catalytic site of the toxin. In some instances, it was even reported that immunity protection uses potent double controls, such as against the ADP-ribosylating toxin (Tre1) of *Serratia proteamaculans* [8]. Not only does immunity block the Tre1 active site, but it also counteracts its activity by removing the ADP that would have been transferred to the FtsZ tubulin that is essential for cell division. In all cases, toxin–immunity pairings help differentiate self from nonself so that only sensitive bacteria without immunity will be intoxicated and killed. This can even be extended to the strain level. For example, within a single species such as *P. aeruginosa*, protection from the DNase Tse7 is provided by the very cognate immunity Tsi7, which means that Tsi7 from a PAK strain will not protect against intoxication by the Tse7 toxin of a PAO1 strain [9].

Whereas T6SS is best known as a contact-dependent antibacterial weapon, recent findings have demonstrated its role in capturing essential nutrients (e.g. manganese) from the environment [10]. In this case, the competitive advantage provided by the T6SS is to restrict resource availability to other microorganisms. This is particularly true for essential ions like iron or manganese. The T6SS releases effector proteins into the surrounding medium that chelate specific ions, which subsequently retro-bind onto a membrane receptor so that the complexed effector ion is internalized and the essential ion captured and used. Importantly, only the bacteria having such specific receptors on their surface will be able to benefit from the external ion source. This concept is reminiscent of the classic production of a siderophore chelator, like pyoverdine in *P. aeruginosa*, which also involves a specific receptor in the outer membrane. In microbial communities, siderophore cheating is a common process where nonsiderophore producers display receptors on their surface to capture siderophores produced by others. This has led to a cheating and cheating resistance arm race, in which the producer releases increasingly specific siderophores [11].

Song and colleagues (2024) discovered an iron-chelating T6SS effector produced by *Yersinia pseudotuberculosis*, which they called TepC. Expression of the *tepC* gene is under control of Fur and iron limitation, which is no surprise, and TepC is specific for Fe^3+^ and has no binding capacity for other ions such as Zn^2+^ or Mn^2+^. Once iron-loaded, Holo-TepC becomes a “public good” and is recaptured by the TonB-dependent receptor TdsR, so that iron can be used. This means that any species in the microbial community possessing a TdsR-like protein, such as *Escherichia coli*, would be able to benefit from this public good, even if not producing TepC. What is most unique and remarkable about this study is the dual domain structure of TepC. Indeed, in addition to the iron-binding domain, TepC displays another domain with DNase activity. Indeed, DNase is a common antibacterial effector delivered by the T6SS and other secretion systems, such as T5SS or T7SS [12]. The challenge for a cheater, having TdsR and yet not producing TepC, would then be to resist the DNase activity. That is where the concept of T6SS toxin/immunity is evident, because TepC is coproduced with its cognate immunity, TipC, which protects from the DNase. Non-TepC producers will not produce TipC, and thus if they take up TepC, they will be eliminated and killed by the DNase activity of TepC before they can benefit from iron acquisition.

Because of the TepC/TipC pairing, Song and colleagues (2024) coin the term “Trojan horse” for the TepC T6SS effector. As described in Ulysse’s Odyssey, if Trojans had looked into the horse’s mouth, they would have detected the threat. Could a TepC cheater be able to counteract the associated DNase activity by acquiring the immunity gene? This is likely to happen, and it would be interesting to investigate whether there may be *tipC*-encoding species that encode *tdsR* while not encoding *tepC* or even the T6SS gene cluster. There is similar precedent within gut microbiota, where *Bacteroidales* members have acquired large sets of T6SS immunity orphan genes [13], which means they are lacking the cognate T6SS toxins. This mechanism, which is called acquired interbacterial defence (AID), may be a rather common ecological adaptation of microorganisms to cope with other species encountered in their vicinity.

It is always exciting to see how evolution shapes microbial life, and the social evolution theory for microorganisms [14] seems to apply here. The T6SS, which was discovered almost 20 years ago, represents a single bacterial system that has adapted to an enormous variety of biological processes, from antibacterial to antieukaryotic and from contact-dependent injection of toxins to release and uptake of public goods. The structure of the T6SS itself is variable, with different classes likely fulfilling different roles [15]. Lastly, the T6SS toxin/effector loading is unconventional, and specialized effectors or “cargos” have been shown to be associated with many different parts of the T6SS nanomachine (e.g. Hcp, VgrG, or PAAR). Yet this novel T6SS discovery by Song and colleagues (2024) is a major advance, representing a reminder from our “microbial friends” on how clever they are in evolving antibacterial strategies. If we continue exploring their genetic and molecular innovations for this very purpose, we will be able to engineer, design, adapt, and implement novel therapeutic strategies in the fight against bacterial pathogens and their associated antimicrobial resistance mechanisms.

## Data Availability

Data sharing not applicable to this article as no datasets were generated or analysed during the current study.
